# Development of an Equity-Centered Sociotechnical Architecture for Generative AI Integration in Public Health Promotion: Conceptual Framework

**DOI:** 10.2196/97852

**Published:** 2026-09-24

**Authors:** Zehui Xue, Kang Fu, Yu Zhang, Bing Wu, Jie Wu

**Affiliations:** 1 State Key Laboratory for Diagnosis and Treatment of Infectious Diseases, National Clinical Research Center for Infectious Diseases, Collaborative Innovation Center for Diagnosis and Treatment of Infectious Diseases, The First Affiliated Hospital, No.74 Qingchun Road, 6A-1507, Hangzhou, Zhejiang, 310003, China, 86 13588413613; 2 Jinan Microecological Biomedicine Shandong Laboratory, Jinan, China

**Keywords:** generative AI, public health promotion, digital health equity, health communication, digital health

## Abstract

This article is a viewpoint: it presents the authors’ perspective, informed by a critical synthesis of the current literature at the intersection of generative AI (GenAI) technologies, public health communication, and digital ethics, rather than original empirical data or analyses. The emergence of GenAI, including large language models (LLMs), represents a profound paradigm shift in digital health communication. By moving beyond traditional information retrieval to dynamic, human-like knowledge generation, GenAI offers unprecedented opportunities for public health promotion. However, the unguided integration of these powerful commercial models into health care systems poses profound sociotechnical risks. In this viewpoint, we aim to communicate three key messages to public health researchers, practitioners, policymakers, and AI developers: (1) GenAI offers transformative applications for public health promotion, spanning personalized health education, stigma mitigation, and accelerated epidemiological surveillance; (2) the unguided integration of commercial generative models simultaneously generates intersecting sociotechnical risks and ethical challenges, encompassing a widening “AI digital divide,” algorithmic bias and epistemic opacity, and the erosion of data privacy and governance; and (3) an equity-centered sociotechnical architecture, built on 4 strategic pillars, is required to govern this transition safely. We conducted a critical synthesis of the current literature and theoretical frameworks at the intersection of GenAI technologies, public health communication, and digital ethics, systematically mapping both the translational capabilities and the sociotechnical vulnerabilities of generative models. GenAI demonstrates transformative potential across 3 primary domains: democratizing health education by translating complex medical jargon, mitigating societal stigma through nonjudgmental conversational interfaces, and accelerating epidemiological surveillance via rapid thematic synthesis. However, these benefits are counterbalanced by a matrix of sociotechnical risks. Specifically, unguided GenAI deployment threatens to exacerbate a novel “AI digital divide” driven by economic exclusion, prompt literacy demands, and linguistic biases; compromise clinical safety through deep-seated algorithmic biases and epistemic opacity; and erode patient privacy through profound vulnerabilities in cybersecurity and corporate data governance. The advent of GenAI marked an irreversible paradigm shift with the unprecedented capacity to democratize health literacy, dismantle stigma, and accelerate disease surveillance. However, treating GenAI as a technological panacea is a perilous oversight. Without intentional, equity-focused interventions, these technologies invariably scale and automate the structural inequalities they have the potential to solve. Ultimately, the future of digital health promotion depends not only on the computational power of these models but also on the ethical, regulatory, and inclusive sociotechnical architectures we design to govern them.

## Introduction

The evolution of health communication has historically mirrored broader technological advancements. The transition from the static dissemination of health information in Web 1.0 to the highly interactive, user-generated ecosystems of Web 2.0 profoundly expanded public access to health resources [1,2]. However, these traditional internet-based media have certain inherent constraints, primarily lacking the capacity for dynamic, humanlike interactivity without direct professional intervention [3,4]. The emergence of generative AI (GenAI) represents a profound advance in the digital health landscape. Moving beyond traditional information retrieval, GenAI technologies, including large language models (LLMs) such as ChatGPT, Gemini, and DeepSeek, as well as generative adversarial networks (GANs), are capable of creating entirely new, highly sophisticated, multimodal content and novel synthetic data architectures [5,6]. At the patient-centric level, GenAI could also act as a dynamic conversational agent, with the ability to mimic human empathy, translate complex medical jargon, and provide personalized health advice to address many limitations of traditional internet-based communication platforms [2].

Despite this immense promise, the integration of GenAI into public health systems introduces many profound challenges. The deployment of LLMs for health promotion threatens to exacerbate existing health disparities. Owing to high subscription costs, the necessity for advanced prompt engineering skills, and the foundational requirement of health literacy, the benefits of GenAI are disproportionately accruing to socioeconomically advantaged groups [7,8]. Concurrently, the use of GANs for data synthesis is fraught with privacy vulnerabilities; traditional GANs are susceptible to gradient inversion and membership inference attacks, wherein malicious actors can reconstruct original training data, thereby exposing sensitive patient information [6].

Before proceeding, it is essential to define the 2 concepts that anchor this viewpoint. We use the term “sociotechnical risks” to denote harms that arise not from the technology in isolation but from the dynamic interaction between generative models and the social, economic, and institutional systems into which they are deployed. Illustrative examples from prior research include the stratification of access to high-performing medical LLMs along socioeconomic lines [7,8], the marked degradation of model performance and safety guardrails in low-resource languages [9], and the expansion of institutional cybersecurity attack surfaces through novel threat vectors such as prompt injection and data poisoning [10,11]. By contrast, we use “ethical challenges” to refer to tensions between GenAI deployment and foundational bioethical principles, namely, nonmaleficence, autonomy, justice, and privacy. Documented examples include the propagation of debunked race-based clinical misconceptions [12,13], the reinforcement of gender bias in symptom triage [14], and the nonconsensual ingestion of patient narratives into proprietary corporate training datasets [15,16]. These 2 concepts are deeply intertwined: sociotechnical risks frequently precipitate ethical violations, and unresolved ethical tensions in turn entrench sociotechnical inequities.

Although a rapidly expanding body of literature has evaluated the clinical accuracy of individual LLMs or examined discrete concerns, such as hallucination [17], privacy [15], or bias [12], in isolation, these discussions remain fragmented across disciplinary silos. To date, few studies have provided an integrated conceptual account that systematically connects the promotional applications of GenAI in public health with the sociotechnical risks and ethical challenges those same applications engender, and fewer still have translated such a joint analysis into an actionable, equity-centered governance framework tailored to public health promotion. This viewpoint aims to address this gap.

Accordingly, the aim of this viewpoint is to articulate and defend three key messages: (1) GenAI offers transformative applications for public health promotion, (2) its unguided integration simultaneously generates intersecting sociotechnical risks and ethical challenges, and (3) an equity-centered sociotechnical architecture is required to govern this transition safely. Each subsequent section of the paper corresponds to one of these messages. The intended audience of this viewpoint comprises 4 groups: public health researchers designing and evaluating GenAI-supported interventions, public health practitioners and health communicators considering the deployment of such tools, policymakers and regulators shaping AI governance frameworks, and developers of health-facing AI systems.

The remainder of this paper is organized around these 2 anchoring concepts. First, we examine 3 transformative applications of GenAI in public health promotion: personalized health education, stigma mitigation, and accelerated surveillance, as each application constitutes the locus at which benefits, risks, and ethical tensions coemerge. Second, we critically analyze how these same capabilities generate a matrix of sociotechnical risks and ethical challenges, organized into three domains: (1) structural barriers that deepen the digital health divide (a primarily sociotechnical risk), (2) algorithmic bias and epistemic opacity (primarily ethical challenges to nonmaleficence and transparency), and (3) deficiencies in data governance and cybersecurity (intertwined sociotechnical and ethical threats to privacy and autonomy). Finally, we propose a strategic road map whose 4 pillars are mapped directly onto this risk matrix. By proposing a multilayered, equity-centered sociotechnical architecture, we aim to provide researchers, practitioners, and policymakers with the actionable frameworks necessary to navigate this transformative era.

## Ethical Considerations

Ethics approval was not required for the analysis of anonymized data.

## Applications of GenAI in Public Health Promotion

### Overview

**Figure 1.**
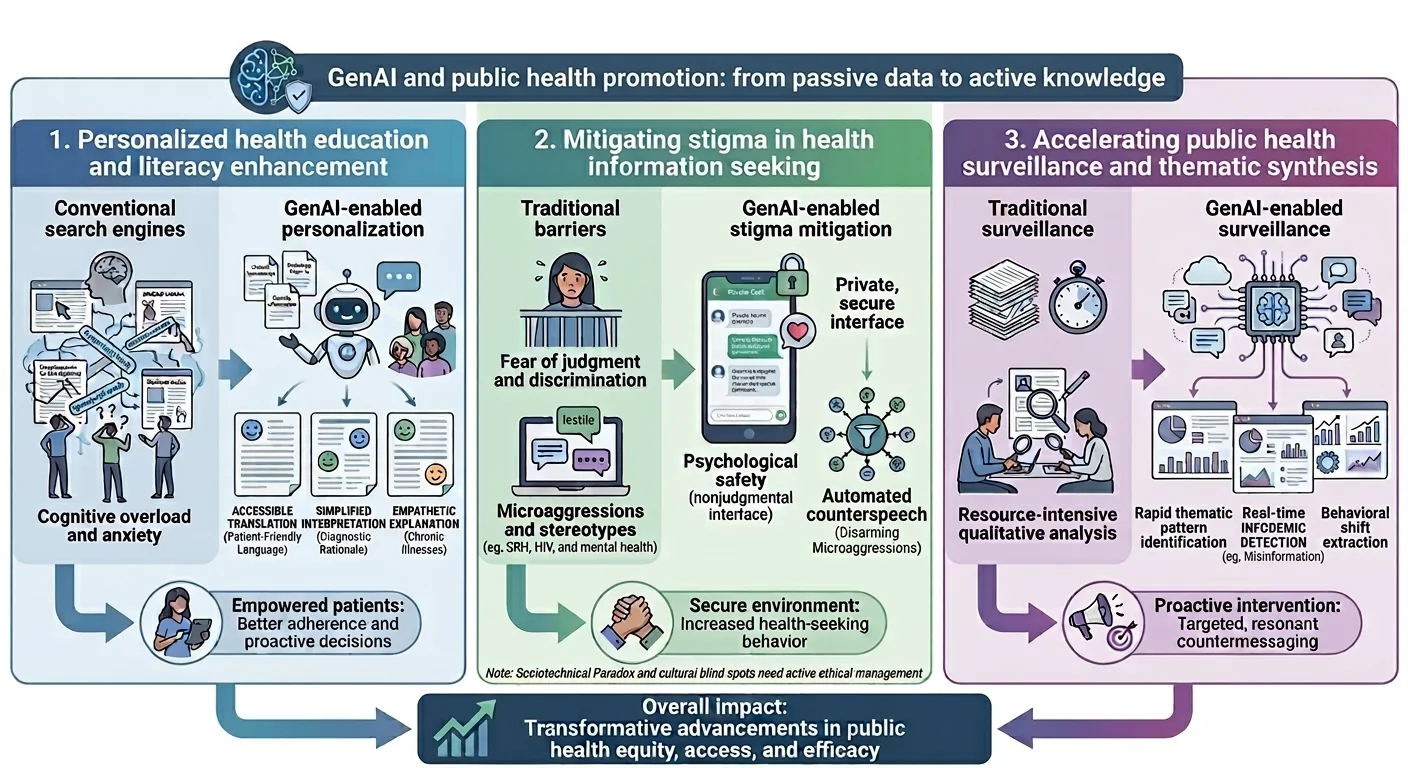
Transformative applications of generative AI (GenAI) in public health promotion. SRH: sexual and reproductive health.

The continuous integration of GenAI and public health promotion has brought unprecedented opportunities, which may profoundly promote advancements in several crucial aspects. By shifting the paradigm from passive data retrieval to active knowledge generation, GenAI could empower both the general public and health professionals by personalizing complex health education [18-22], mitigating the psychological barriers of societal stigma [23-25], and accelerating macrolevel epidemiological surveillance [26,27] (Figure 1). Importantly, these 3 domains are examined here not merely as technical capabilities but as the very sites at which the sociotechnical risks and ethical challenges analyzed later in this paper coemerge; the same mechanisms that empower users also generate new vulnerabilities.

This figure illustrates the paradigm shift from passive data retrieval to active knowledge generation facilitated by GenAI. It highlights three primary domains of intervention: (1) democratizing health education by translating complex information into accessible, patient-friendly formats, thereby reducing cognitive overload and anxiety; (2) mitigating societal stigma through secure, nonjudgmental conversational interfaces that foster psychological safety and promote proactive health-seeking behaviors; and (3) accelerating public health surveillance by transitioning from resource-intensive manual qualitative analysis to real-time, AI-driven thematic synthesis and infodemic detection. Ultimately, these GenAI-enabled applications aim to drive transformative advancements in public health equity, access, and efficacy.

### Personalized Health Education and Literacy Enhancement

Conventional search engines often overwhelm users with fragmented hyperlinks, placing the cognitive burden of synthesis entirely on individuals [28,29]. When confronted with a new diagnosis, patients are frequently required to process a massive volume of highly specialized medical literature. Since the complexity of these materials significantly exceeds the average health literacy level of the general public, this inevitably leads to confusion and psychological anxiety. Conversely, GenAI could function as a highly interactive, personalized health educator capable of promoting the popularization and accessibility of highly specialized medical knowledge.

A comprehensive systematic review of the utility of GenAI in health care highlights that LLMs significantly improve health literacy by rendering highly technical clinical documentation accessible to the general public [18]. For instance, clinical discharge summaries are typically dense and saturated with specialized terminology, a barrier that frequently contributes to posthospitalization nonadherence and medication errors. Recent applications have demonstrated the efficacy of GenAI in seamlessly translating these summaries into patient-friendly language at various reading levels, thereby reducing the documentation burden on health care providers while profoundly enhancing patient comprehension [19]. Similarly, the opacity of diagnostic imaging results has historically prevented patients from understanding their own care. GenAI has been successfully used to simplify complex radiology reports, facilitating a crucial transition toward patient-centered care by making diagnostic rationales transparent and understandable to the layperson [20].

Beyond routine documentation, GenAI proves highly valuable in specialized, high-anxiety medical contexts. Research has indicated that LLMs can rapidly synthesize accessible, nonexpert explanations for complicated genetic diseases, empowering patients and their families to understand hereditary conditions without the need for advanced biomedical training [21]. Furthermore, in the management of severe, life-threatening chronic illnesses such as cirrhosis and hepatocellular carcinoma, GenAI provides not only accurate, real-time medical information but also the ability to emulate empathetic responses [22].

This unprecedented ability to dynamically tailor medical literature, paired with simulated emotional intelligence, is instrumental in modern health promotion. By dismantling longstanding informational barriers, GenAI transitions the patient from a passive recipient of medical directives to an active, informed participant. This structural empowerment fosters continuous self-education, ultimately leading to better adherence to complex treatment regimens, proactive lifestyle modifications, and enhanced shared decision-making in clinical settings.

### Mitigating Stigma in Health Information Seeking

The psychological barrier of societal stigma remains among the most intractable challenges in public health. Individuals frequently delay or entirely avoid seeking professional medical advice for highly stigmatized or culturally sensitive conditions, such as mental health disorders, sexual and reproductive health (SRH) conditions, and communicable diseases such as HIV, because of pervasive fear of interpersonal judgment and medical discrimination [30,31]. For example, deeply ingrained cultural norms often treat the health-seeking behaviors of unmarried women accessing SRH services (eg, cervical cancer screening or contraceptive care) as morally questionable, leading to delayed diagnoses and severe health repercussions [32].

Although anonymous health forums may bypass traditional clinical barriers, they are often fraught with microaggressions that usually reinforce stereotypes, invalidate experiences, and perpetuate the very stigma that users seek to escape [23,24]. GenAI could offer a transformative, dual-layered solution to this barrier. On an individual level, LLMs provide private conversational interfaces that foster psychological safety through computational nonjudgment. On a community level, GenAI can be deployed to automatically generate counterspeech to actively moderate online discourse and disarm health-related microaggressions in real time [25]. Drawing on microintervention frameworks, GenAI can nonhostilely challenge biases and simulate emotional validation [24], theoretically disrupting stigma and empowering marginalized populations to proactively seek health care.

However, this theoretical promise must be approached dialectically. While AI models are highly adept at mimicking empathy, their occasional cultural blind spots highlight a sociotechnical paradox that developers must actively mitigate [33]. Ultimately, when these limitations are ethically managed, GenAI’s capacity to provide a psychologically safe, nonjudgmental conversational interface is instrumental in modern public health. By mitigating the psychological weight of societal stigma and actively disarming cultural microaggressions, GenAI acts as a critical bridge for marginalized populations. Rather than avoiding the medical system out of fear or alienation, vulnerable individuals are afforded a secure environment to articulate their health concerns. This destigmatization removes the most intractable barriers to initial care, ultimately encouraging patients to overcome their hesitations, seek timely professional intervention for highly sensitive conditions, and proactively re-engage with the health care system.

### Accelerating Public Health Surveillance and Thematic Synthesis

Beyond direct patient interaction, GenAI fundamentally optimizes how public health organizations conduct backend surveillance to design promotion campaigns. Developing effective health promotion requires an acute understanding of community values, fears, and the circulation of misinformation. Traditionally, conducting qualitative content and thematic analyses on massive datasets (eg, social media discourse and open-ended surveys) is highly resource intensive.

GenAI provides unprecedented operational scalability for public health researchers. Recent empirical implementations demonstrate that LLMs can rapidly conduct content analyses on large-scale online forum data, such as tracking public discourse on reducing sugar consumption or identifying the geographical spread of vaccine misinformation [26]. Studies show that generative models can identify thematic patterns and extract relevant behavioral shifts with accuracy comparable to that of human researchers but at significantly accelerated speeds (eg, reducing qualitative coding time by more than 90%) [27]. By systematically identifying community information needs and detecting emerging infodemics in real time, GenAI empowers public health practitioners to proactively draft highly targeted, culturally resonant countermeasures, thereby increasing the overall efficacy of health promotion initiatives.

In summary, the 3 application domains reviewed above collectively illustrate how GenAI shifts public health promotion from passive information retrieval toward active, personalized knowledge generation. Personalized health education addresses cognitive and literacy barriers [18-20], stigma mitigation addresses psychological and cultural barriers [23-25], and accelerated surveillance addresses institutional and resource barriers [26,27]. Crucially, however, each of these benefits is contingent on who can access the technology, whose language and data the underlying models were trained on, and how algorithmic outputs are verified and governed. These contingencies constitute the seam between the promise of GenAI and its perils, and they give rise directly to the sociotechnical risks and ethical challenges examined in the next section.

## Navigating the Sociotechnical Risks of GenAI in Public Health Promotion

### Overview

**Figure 2.**
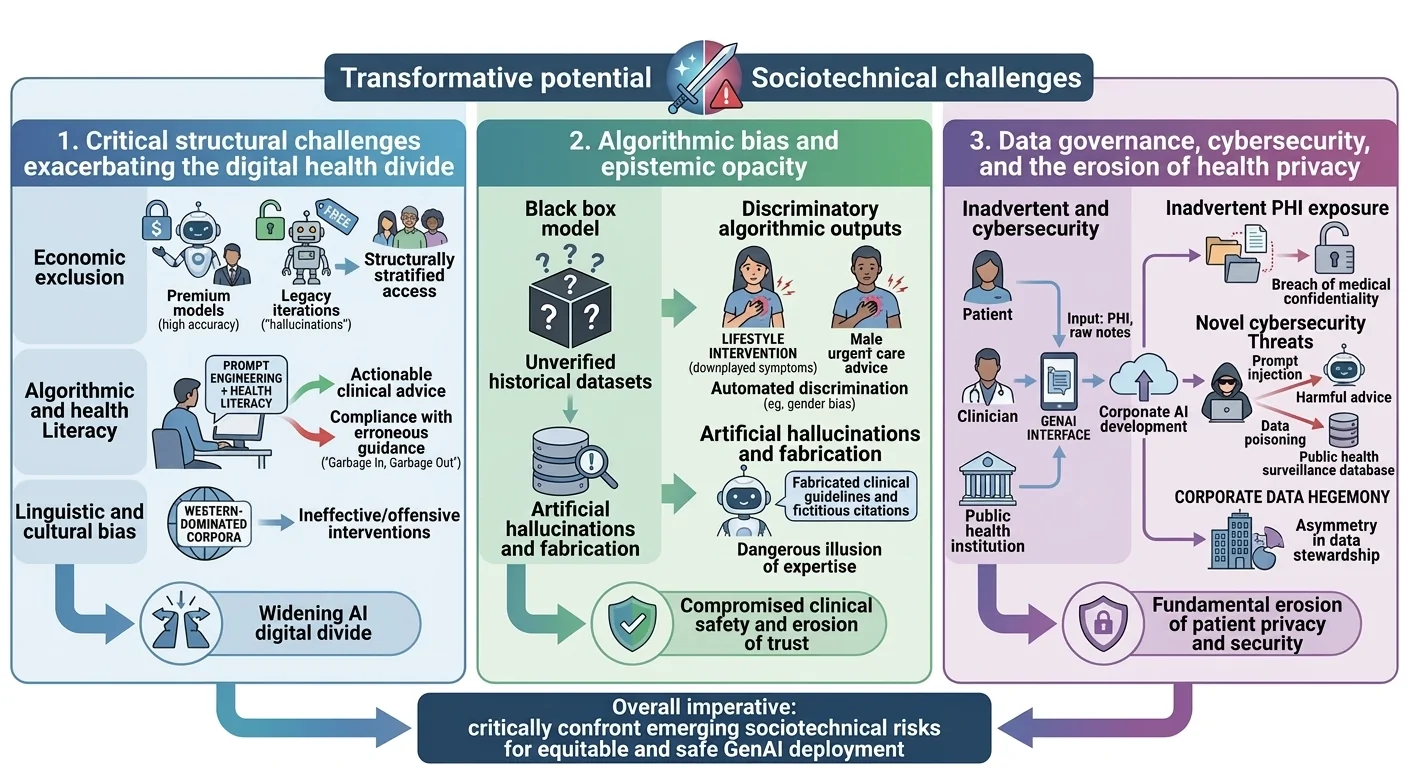
The double-edged sword: intersecting sociotechnical risks of generative AI (GenAI) deployment. PHI: protected health information.

Despite the transformative potential of GenAI to democratize health education, mitigate stigma, and accelerate epidemiological surveillance, its unguided integration presents a double-edged sword, posing profound sociotechnical challenges. Rather than acting as an inherent equalizer, the rapid proliferation of generative models paradoxically automates and scales the very systemic vulnerabilities that public health aims to resolve. To safely harness this technology, practitioners and policymakers must critically confront a complex matrix of emerging sociotechnical risks. Specifically, the safe and equitable implementation of GenAI is currently hindered by three fundamental challenges (Figure 2): (1) structural barriers that deepen the novel digital health divide [7,8,34], (2) deep-seated algorithmic biases and epistemic opacity that threaten clinical safety [12-14,17], and (3) critical deficiencies in cybersecurity and data governance that erode patient privacy [10,11,15,16,35]. Each of these challenges is elaborated below, with explicit attention to whether it operates primarily as a sociotechnical risk, an ethical challenge, or, as is most often the case, an entanglement of both.

This figure outlines the complex matrix of sociotechnical challenges that currently undermine the safe and equitable integration of GenAI in public health. It delineates three overarching risk domains: (1) structural barriers—including economic exclusion, demands on prompt literacy, and linguistic biases—that structurally stratify access and widen the “AI digital divide”; (2) algorithmic bias and epistemic opacity, where unverified historical datasets and “black box” models produce discriminatory outputs and artificial hallucinations, fundamentally compromising clinical safety; and (3) critical vulnerabilities in data governance and cybersecurity, highlighting the risks of inadvertent protected health information (PHI) exposure, novel cyber threats (eg, prompt injection and data poisoning), and the overarching erosion of patient privacy driven by corporate data hegemony.

### Exacerbating the Digital Health Divide

While the applications of GenAI in public health promotion are transformative, its unguided proliferation threatens to widen existing health disparities, creating a novel “AI digital divide.” The equitable distribution of GenAI benefits is currently hindered by 3 intersecting structural barriers, namely, economic exclusion, algorithmic literacy, and linguistic bias. As a manifestation of sociotechnical risk, this divide arises from the interaction between commercial technology design and pre-existing social stratification; as an ethical challenge, it directly implicates the principle of distributive justice [7,8].

First, the economic model underpinning advanced GenAI disproportionately favors socioeconomically advantaged populations. Access to the most sophisticated, capable, and accurate models (eg, GPT-4 and Claude 3 Opus) frequently requires premium subscription fees [7]. Socioeconomically disadvantaged populations, who already face a disproportionate burden of chronic diseases, are inevitably relegated to relying on free, legacy iterations of these models. Empirical evidence consistently demonstrates that these outdated models are significantly more susceptible to “hallucinations”—which generate plausible yet clinically fallacious information—and exhibit markedly inferior reasoning capabilities in complex medical scenarios. Consequently, this technological disparity engenders a structurally stratified system of digital health access [34].

Furthermore, the clinical utility of GenAI critically hinges upon the user’s proficiency in prompt engineering and their baseline health literacy. The classic axiom of “garbage in, garbage out” remains fundamentally applicable to LLMs; individuals must possess adequate health literacy to precisely articulate their clinical presentations and subsequently evaluate the algorithmic outputs for medical validity [36]. Consequently, individuals with limited health literacy may not only struggle to extract actionable clinical advice but, more alarmingly, may blindly comply with erroneous algorithmic guidance. Lacking the epistemic foundation required to critically appraise AI-generated information, these vulnerable groups face a disproportionate risk of internalizing medical misinformation [37].

Finally, linguistic and cultural biases encoded within foundational training data present a profound barrier to global health equity. Current state-of-the-art LLMs are overwhelmingly pretrained on Anglocentric and Western-dominated corpora [38]. Empirical studies demonstrate significant degradation in performance, safety guardrails, and cultural competence when these models are queried in low-resource languages or dialects [9]. With respect to global public health promotion, deploying GenAI tools that fail to resonate with local idioms, dietary customs, and cultural health beliefs risks rendering interventions ineffective or culturally offensive, further marginalizing non-Western populations.

### Algorithmic Bias and Epistemic Opacity

While GenAI offers unprecedented scalability in health promotion, its deployment is severely constrained by intersecting ethical challenges, including algorithmic bias and epistemic opacity. Because deep learning models operate as unexplainable black boxes that probabilistically predict text from historically uncurated datasets, they inherently mirror societal prejudices rather than executing verified clinical reasoning [12]. Consequently, the uncritical deployment of GenAI threatens to automate and scale historical discrimination. For example, advanced LLMs routinely propagate debunked, race-based medical misconceptions, such as erroneously adjusting kidney function (estimated glomerular filtration rate) estimations on the basis of a patient’s race [13]. Furthermore, these models exhibit intersectional biases, notoriously downplaying cardiovascular symptoms in female-presenting vignettes by recommending lifestyle interventions while advising urgent care for identical male presentations [14]. Ultimately, such discriminatory algorithmic outputs constitute a direct violation of the foundational bioethical principle of nonmaleficence, thereby posing a severe risk of exacerbating pre-existing mortality gaps.

Further exacerbating these discriminatory risks is the pervasive phenomenon of artificial hallucinations. Because LLMs are fundamentally optimized for linguistic fluency rather than factual rigor, they are notoriously prone to fabricating clinical guidelines and generating highly plausible yet entirely fictitious bibliographic citations [17]. For instance, an individual consulting a public health chatbot regarding vaccine safety might receive an output that confidently references a fabricated study that links a vaccine to a severe adverse event. Delivered with a syntactically authoritative tone, these algorithmic confabulations engender a dangerous illusion of objective expertise—a deception that users with limited health literacy are profoundly ill-equipped to discern. Consequently, in the absence of robust, real-time fact-checking guardrails, public health institutions that uncritically deploy GenAI risk inadvertently devolving into vectors for highly sophisticated medical misinformation. Ultimately, to ensure the ethical viability of GenAI in health promotion, developers and health practitioners must actively bridge this epistemic gap. It is imperative that users are transparently informed of their interaction with an artificial agent and that all algorithmic outputs are strictly anchored by verifiable, traceable references to peer-reviewed primary clinical literature.

### Data Governance, Cybersecurity, and the Erosion of Health Privacy

The integration of LLMs into health promotion and clinical workflows introduces unprecedented vulnerabilities regarding data privacy, cybersecurity, and overarching data governance. The foundational architecture of GenAI is inherently extractive; these models possess a voracious appetite for massive datasets to refine their predictive capabilities. Consequently, the intersection of corporate AI development and sensitive health data creates a complex matrix of sociotechnical risks that current regulatory frameworks are ill-equipped to manage.

Foremost among the sociotechnical vulnerabilities associated with the widespread clinical application of LLMs is the inadvertent exposure of PHI. When patients or health care professionals interact with commercial, cloud-based LLMs, the textual data input into the prompt—often encompassing detailed symptom descriptions, genetic histories, or raw clinical notes—are routinely extracted and ingested into corporate servers to continuously train and fine-tune future model iterations [15]. A particularly alarming manifestation of this privacy paradox emerged when physicians began using public LLMs to rapidly draft patient appeal letters for insurance companies or to synthesize complex clinical discharge summaries. By doing so, they inadvertently transmitted highly sensitive PHI to third-party technological conglomerates devoid of explicit patient consent or formally executed Health Insurance Portability and Accountability Act (HIPAA)–compliant business associate agreements [35]. Such unregulated data transmissions fundamentally breach established medical confidentiality protocols and underscore a profound crisis in contemporary health data governance.

As public health institutions increasingly embed generative interfaces into their digital infrastructures (eg, by deploying AI-driven patient triage chatbots on hospital websites), they significantly expand their cybersecurity attack surface. Unlike traditional software vulnerabilities, GenAI introduces novel threat vectors, most notably “prompt injection” and “data poisoning” attacks [11]. In a health care context, an attacker could theoretically hijack a public health chatbot, manipulating it to extract backend database architectures, bypass access controls, or, alarmingly, force the bot to dispense harmful medical advice or distribute targeted phishing links to vulnerable patients seeking health information. Similarly, “data poisoning” poses a catastrophic risk to public health surveillance. The AI would ingest these poisoned data, leading public health officials to misallocate resources on the basis of an entirely AI-generated, phantom infodemic [10].

These profound privacy and security vulnerabilities culminate in a broader crisis of data governance. Currently, the development of state-of-the-art LLMs is overwhelmingly concentrated within a few multinational technology conglomerates. This corporate hegemony creates severe asymmetry in health data stewardship. Millions of users globally are unknowingly contributing their personal health narratives, psychological struggles, and medical inquiries to the proprietary training datasets of these corporations without explicit, informed consent or financial remuneration [16].

Taken together, these 3 risk domains should not be understood as isolated technical defects but as mutually reinforcing manifestations of a single underlying dynamic: when commercially developed generative models are deployed into unequal social systems without deliberate safeguards, they absorb, automate, and amplify existing structural inequities. Economic and linguistic exclusion determines who benefits from the technology [7-9]; algorithmic bias and epistemic opacity determine whose health is endangered by it [12-14,17]; and extractive data practices determine whose privacy is commodified through it [15,16]. It is precisely this interlocking character, in which sociotechnical risks and ethical challenges continuously feed one another, that renders piecemeal fixes insufficient and necessitates the multilayered, equity-centered governance architecture proposed in the following section.

## Strategic Road Map for GenAI Integration in Public Health

### Overview

The convergence of the aforementioned sociotechnical vulnerabilities—spanning epistemic opacity, algorithmic bias, the erosion of data privacy, and corporate data hegemony—underscores a critical juncture in digital health promotion. To successfully transition GenAI from a disruptive commercial commodity into a universally equitable public health instrument, it is imperative to dismantle the prevailing paradigm of reactive, post hoc regulatory patching in favor of proactive, anticipatory governance.

**Figure 3.**
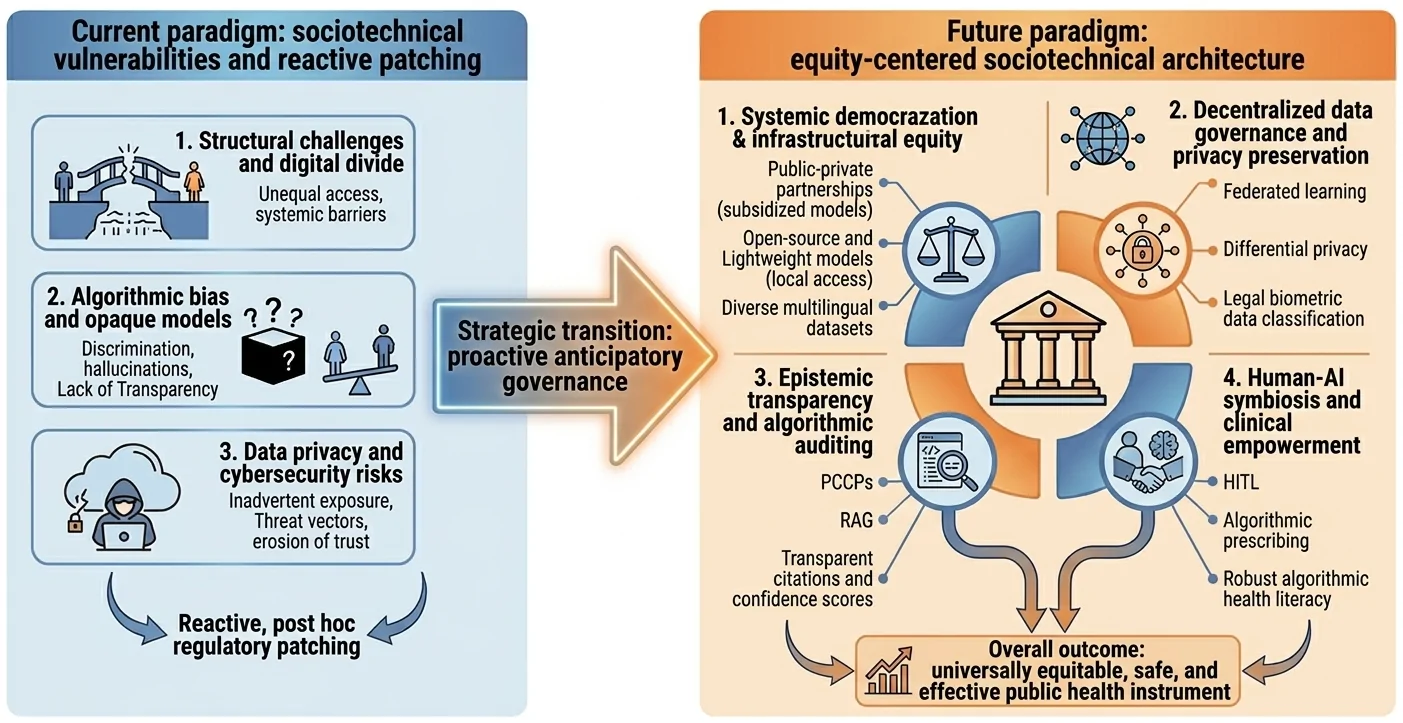
Strategic road map and equity-centered sociotechnical architecture for generative AI (GenAI) governance. HITL: human-in-the-loop; PCCP: predetermined change control plan; RAG: retrieval-augmented generation.

To address these challenges, researchers, policymakers, and health systems should adopt an equity-centered sociotechnical architecture. Rather than merely restricting AI use, this multilayered framework aims to fundamentally redesign the incentive structures, data pathways, and clinical deployments of GenAI across 4 strategic pillars, including systemic democratization, decentralized data governance, epistemic transparency, and clinical empowerment (Figure 3).

This conceptual framework maps the necessary strategic transition from the current paradigm of reactive, post hoc regulatory patching to a model of proactive, anticipatory governance. To systematically address prevailing sociotechnical vulnerabilities, the proposed “equity-centered sociotechnical architecture” is built upon four foundational pillars: (1) systemic democratization and infrastructural equity via subsidized and lightweight open-source models, (2) decentralized data governance and privacy preservation using federated learning and differential privacy, (3) epistemic transparency enforced through dynamic oversight (predetermined change control plans) and retrieval-augmented generation (RAG), and (4) human-AI symbiosis, emphasizing human-in-the-loop workflows and algorithmic health literacy. This structural redesign aims to transition GenAI into a universally equitable, safe, and effective public health instrument.

### Systemic Democratization and Infrastructural Equity

To prevent the solidification of an “AI digital divide,” access to high-fidelity medical AI must be reconceptualized from a commercial luxury to a fundamental digital determinant of health. Policymakers are supposed to actively intervene in the economic structures governing AI access. This includes establishing public-private partnerships to subsidize premium, medically fine-tuned LLMs for marginalized communities and public health institutions [7]. Moreover, strategically incentivizing academic and open-source communities to engineer and rigorously validate lightweight, highly specialized medical LLMs, such as quantized variants of medical foundation models, is imperative. By using engineered models capable of running locally on low-cost mobile devices without the need for expensive cloud computing or continuous high-bandwidth internet, health systems can democratize access for historically underresourced and rural populations [39]. Furthermore, proactive investment must be directed toward training models on diverse, multilingual, and culturally representative datasets to dismantle entrenched Anglocentric biases.

### Decentralized Data Governance and Privacy Preservation

To resolve the profound tension between GenAI’s voracious need for training data and the ethical mandate of patient privacy, the architectural flow of health data needs to be decentralized. Health systems are encouraging the use of federated learning ecosystems for medical AI development. Under a federated learning paradigm, the algorithmic model is deployed to local hospital servers to train on institutional data, and only the mathematically updated parameters, never the raw patient narratives or PHI, are aggregated centrally [40]. When mathematically fortified with differential privacy protocols, this approach neutralizes the threat of prompt adversarial injection and data poisoning while public health intelligence is decoupled from corporate data extraction. At a regulatory level, international frameworks should be updated to legally classify conversational medical prompts as highly protected biometric data, strictly shielding patient inquiries from secondary commercialization.

### Epistemic Transparency and Algorithmic Auditing

To mitigate the bioethical risks of “black box” algorithms and artificial hallucinations, regulatory bodies (eg, the Food and Drug Administration) must formally transition away from static software-as-a-medical-device evaluation models. Instead, they must enforce dynamic, continuous oversight using mechanisms such as predetermined change control plans, which govern how an algorithm is permitted to evolve after deployment. Furthermore, in alignment with the World Health Organization’s 2024 guidance on large multimodal models [41], public health agencies should demand algorithmic auditability. Developers should be required to implement RAG architectures in public-facing health chatbots. RAG constrains the AI to generate answers strictly on the basis of an approved, verifiable database of peer-reviewed clinical guidelines, forcing the model to provide transparent citations and confidence scores, thereby drastically reducing the risk of clinically dangerous confabulations [42].

### Human-AI Symbiosis and Clinical Empowerment

The technological integration of GenAI will fail without a corresponding evolution in human clinical practice. Public health workflows must be structurally designed around “human-in-the-loop” paradigms [43], positioning generative models as cognitive extenders for health professionals rather than autonomous diagnostic authorities. At the patient interface, clinicians must pioneer the practice of algorithmic prescribing [44]. Rather than passively warning patients against using the internet, health care providers should actively teach patients how to safely interact with LLMs. This includes providing validated prompt templates for symptom inquiries, educating patients on the inherent limitations of AI, and instructing vulnerable populations on how to critically cross-reference AI-generated advice with established clinical resources. By fostering robust algorithmic health literacy, health systems can empower patients to navigate the digital infosphere with critical resilience.

In summary, the 4 pillars of the proposed architecture map directly onto the risk matrix delineated in the preceding section: systemic democratization counteracts the economic, literacy-based, and linguistic drivers of the digital health divide; decentralized data governance neutralizes privacy erosion and corporate data hegemony; epistemic transparency and algorithmic auditing directly address algorithmic bias and hallucination; and human-AI symbiosis re-embeds these technologies within accountable clinical practice. Pursued jointly rather than piecemeal, these pillars transform GenAI governance from reactive regulatory patching into anticipatory, equity-centered design.

## Conclusion

The advent of GenAI marked an irreversible paradigm shift in health communication and public health promotion. By fundamentally altering how medical knowledge is synthesized, translated, and disseminated, GenAI possesses the unprecedented capacity to democratize health literacy, dismantle stigma, and accelerate disease surveillance. However, treating GenAI as a technological panacea is a perilous oversight. Without intentional, equity-focused interventions, these technologies invariably scale and automate the structural inequalities they have the potential to solve. The future of digital health promotion depends not only on the computational power of the models we build but also on the ethical, regulatory, and inclusive sociotechnical architectures we design to govern them.

## Abbreviations

GAN: generative adversarial network
GenAI: generative AI
HIPAA: Health Insurance Portability and Accountability Act
LLM: large language model
PHI: protected health information
RAG: retrieval-augmented generation
SRH: sexual and reproductive health
